# The Improvement of Physical Function and Caregiver Burden by a Multimodal Intervention: A Case Study of Combined Exercise Therapy, Nutritional Guidance, and Hydrogen Gas Inhalation Therapy

**DOI:** 10.7759/cureus.79516

**Published:** 2025-02-23

**Authors:** Yuusuke Harada, Ryoko Okamura, Junpei Sawano, Nao Koide, Michiko Miyakawa

**Affiliations:** 1 Graduate School of Humanities and Social Sciences, Hiroshima University, Hiroshima, JPN; 2 Graduate School of Medicine, Chiba University, Chiba, JPN; 3 Department of Rehabilitation, Asahikawa Medical University Hospital, Asahikawa, JPN; 4 Department of Rehabilitation, Hokusei Hospital, Hokkaido, JPN; 5 Department of Rehabilitation, Sapporo Medical University, Sapporo, JPN; 6 Department of Social Welfare, Niigata University of Health and Welfare, Niigata, JPN; 7 Faculty of Human Environment, Hosei University, Tokyo, JPN

**Keywords:** hydrogen gas inhalation therapy, multimodal intervention, nutrition guidance, sarcopenia, therapeutic exercise

## Abstract

The objective of this exploratory case study was to examine the impact of a multifaceted intervention, which incorporated exercise therapy, nutritional guidance, and hydrogen gas inhalation, on the physical function and caregiver burden of an older female patient suspected of having sarcopenia. The methods employed included a three-month program of group exercise and individualized exercise sessions, three times per week, in addition to nutritional guidance and hydrogen gas inhalation therapy. The primary outcome measures included grip strength, walking speed, inflammatory/oxidative stress markers (c-reactive protein (CRP), interleukin-6 (IL-6), 8-hydroxy-2' -deoxyguanosine (8-OHdG)), and caregiver burden, which was assessed by the Family Caregiver Burden Scale (FCS). The results demonstrated that after a period of three months, there was an improvement in grip strength and walking speed. Concurrently, there was a decrease in CRP, IL-6, and 8-OHdG levels. The FCS score demonstrated a shift from the "severe" range to the "normal" range, suggesting a reduction in caregiver burden. The findings of this case study suggest that a multidisciplinary, multifaceted intervention combining exercise therapy, nutritional support, and hydrogen gas inhalation may be effective in enhancing physical function and reducing caregiver burden in older adults with suspected sarcopenia. However, further research is necessary to clarify the independent effects of hydrogen gas inhalation.

## Introduction

Sarcopenia is a multifactorial disease associated with aging, primarily characterized by the loss of skeletal muscle mass and function among older adults [1,2]. First proposed as a concept in 1997 [3], sarcopenia has been shown to influence the prognosis of various diseases even in Japan, which boasts the world's highest life expectancy [4]. 

The underlying mechanisms of sarcopenia have been elucidated to include deterioration of the neuromuscular junction, an imbalance in protein synthesis and degradation caused by chronic inflammation, and functional impairments of satellite cells [5]. Accelerating factors such as hormonal changes, increased inflammation, oxidative stress, mitochondrial dysfunction, and autophagy abnormalities [2] have also been identified. These processes are thought to lead to a reduction in muscle fiber number and cross-sectional area, as well as a decrease in muscle quality, ultimately resulting in a decline in muscle strength and functional capacity [6]. 

Although adequate nutrition, habitual exercise, and active physical activity are recommended for the prevention of sarcopenia [7], knowledge regarding treatment remains limited. While resistance training has been suggested to increase muscle mass and strength in older adults [6], its long-term outcomes are still unclear. Consequently, there is an imperative for further accumulation of evidence on treatment effectiveness to facilitate the development of targeted preventive strategies and identify potential therapeutic interventions aimed at enhancing the quality of life among the elderly [8]. Meanwhile, hydrogen gas has been reported to function as a selective antioxidant by reducing oxidative stress and inflammation in the brain [9]. 

The utilization of hydrogen inhalation therapy, employing hydrogen gas, has been explored in the treatment of a wide range of diseases. A study by Dragana et al. examined the effects of a six-month intervention involving the consumption of hydrogen-rich water in elderly patients over the age of 70 [10]. The findings indicated that hydrogen-rich water led to an approximate 4% increase in mean telomere length, exhibited a tendency to enhance DNA methylation, and demonstrated a substantial improvement in chair-stand capacity. These findings suggest that hydrogen gas may be recognized as an anti-aging agent with the potential to address several features of aging, including loss of function and shortened telomere length. In addition, hydrogen inhalation therapy has shown potential in treating ischemia-reperfusion injury, myocardial infarction, and organ transplantation [11]. The underlying mechanisms of hydrogen inhalation therapy have been elusive, but recent studies have begun to elucidate its potential. In vitro and in vivo studies have suggested that hydrogen gas, when inhaled, may reduce proinflammatory cytokines and pro-apoptotic factors. Additionally, modulation of microRNA expression and activation of various signaling pathways related to angiogenesis and cellular protection have been observed [12]. These findings have led to renewed interest in the use of hydrogen inhalation therapy as a rehabilitation modality for patients recovering from severe acute respiratory syndrome coronavirus 2 (SARS-CoV-2) infection or ischemic stroke. The available literature suggests that this therapy may lead to improvements in respiratory function, physical capacity, and neurological outcomes. In particular, studies have reported significant increases in walking distance and vital capacity in patients with post-acute sequelae of SARS-CoV-2 infection who underwent hydrogen inhalation therapy [13], along with a reduction in the incidence of asymptomatic hypoxemia and endothelial dysfunction [14]. In stroke patients, hydrogen inhalation therapy has been shown to yield superior efficacy in enhancing neurological scores and MRI signal intensity when compared with conventional treatment methods [15]. The therapeutic effects of this therapy are believed to be attributable to its antioxidant, anti-inflammatory, and anti-apoptotic properties [9]. Moreover, a multitude of studies have reported elevated levels of inflammatory cytokines in sarcopenia cases [16-18], with these increases correlating with reduced skeletal muscle mass [19-20]. In light of these observations, the potential of hydrogen inhalation therapy, with its established anti-inflammatory properties, to address sarcopenia remains a promising avenue for future research. However, to date, there have been no clinical trials or research reports on this topic in Japan or internationally. Currently, various clinical trials on hydrogen inhalation therapy are ongoing, with a focus on controlling aging-related factors in older adults as well as on hypertension and improvements in exercise performance [10,21-22]. In Japan, a study demonstrated that hydrogen inhalation therapy had beneficial effects on reducing oxidative stress and C-reactive protein (CRP) levels in patients undergoing hemodialysis [23]. Nevertheless, large-scale studies and those employing appropriate control groups remain insufficient, making it difficult to conclusively establish the effects attributable solely to hydrogen inhalation therapy. The accumulation of further evidence based on robust scientific data and expanded case numbers in Japan is therefore required.

In consideration of the aforementioned context, the present report delineates a preliminary investigation intended to inform future controlled trials. The objective of this case study is also to examine the potential indications for hydrogen inhalation therapy for sarcopenia. Specifically, one patient with suspected sarcopenia was enrolled in a day rehabilitation program where hydrogen inhalation therapy was combined with a standard program consisting of exercise therapy and nutritional guidance for a three-month intervention period. It is important to note that multiple interventions were conducted in parallel during this period, precluding the isolation of the effect of hydrogen inhalation therapy alone. Nevertheless, the content of this study was a necessary process to confirm the future potential of hydrogen inhalation therapy.

## Case presentation

The patient was a woman in her early 80s who presented with the chief complaint of difficulty in walking. Approximately four months prior to the start of daytime rehabilitation, she fell at home and sustained bruises on her head and left upper arm. Her usual lifestyle consisted of general living, with care provided by her husband, and her main mobility was only in the indoor environment, with very infrequent outings. Following a referral from her primary care provider, she applied for long-term care insurance and began day-care rehabilitation three times per week to reduce her husband's caregiving burden and improve her physical function. The patient had a history of hypertension and heart failure, both of which were followed up during regular outpatient visits. However, the results of blood tests revealed elevated inflammatory markers and oxidative stress, with no clear explanations for these findings. The patient was classified as requiring support level 1, and she resided with her husband, who served as her primary caregiver. Her mobility, both indoor and outdoor, required the use of a T-cane and mild assistance, and household tasks such as meal preparation were performed by her husband or a daughter who resided nearby. The Family Caregiver Burden Scale (FCS) was utilized to assess the main caregiver's burden, with a score of 31 out of 40 points indicating a severe burden.

Pre-intervention investigation

Physical Findings

The participant was 152.4 cm tall, weighed 41.1 kg, and had a BMI of 17.7 kg/m². No communication problems were noted; the Hasegawa Dementia Scale-Revised (HDS-R) score was 25/30, with failure in orientation, digit span retraction, and object recall. Resting blood pressure was 104/74 mmHg and heart rate 62/minute; after a six-minute walk speed test, blood pressure increased to 128/82 mmHg and heart rate to 84/minute.

Laboratory Findings

Laboratory test results showed CRP of 1.2 mg/dL, plasma brain natriuretic peptide (BNP) of 43.2 pg/dL, interleukin-6 (IL-6) of 8.4 pg/mL, serum albumin of 3.6 g/dL, and 8-hydroxy-2′-deoxyguanosine (8-OHdG) of 0.46 ng/mL. These results were indicative of inflammation, but heart failure was well controlled.

Physical Therapy Evaluation

Lower extremity circumference was 31.2 cm, knee extensor strength was grade 4 by manual muscle testing (MMT), and grip strength was 16.5 kg on the left and 17.2 kg on the right. The patient walked for six minutes with a T-cane at a comfortable speed of 0.6 m/s; at baseline, she could not test from sitting to standing due to fear. Bioelectrical impedance analysis (InBody770; InBody Co., Ltd, South Korea) revealed a skeletal muscle mass index (SMI) of 5.5 kg/m² according to the Asian Working Group for Sarcopenia 2019 (AWGS2019) criteria [24], suggesting decreased muscle strength and muscle mass and physical capacity. Gait analysis showed decreased toe clearance at initial contact, resulting in a shuffling gait. In the forehead plane, trunk sway to the side of the stance limb was evident. In the sagittal plane, trunk flexion persisted and hip extension at the stance end was inadequate; a timed up-and-go (TUG) test under comfortable conditions with a T-cane took 17.4 seconds. This test consisted of measuring the time to rise from a chair with a call, go around a target 3 meters away, and sit back in the chair.

Intervention process

The intervention was conducted at a day-care rehabilitation facility, where exercise therapy is routinely provided, with additional input from collaborating researchers in geriatric care, nutrition science, and hydrogen-based therapies.

At the time our intervention commenced, approximately three months after the start of day-care rehabilitation, an evaluation following the AWGS2019 criteria revealed low muscle mass, as well as low muscle strength and physical performance as mentioned above. Although referral for medical treatment was recommended, she did not receive a definitive sarcopenia diagnosis due to her family's preference to continue care at the day-care rehabilitation facility. In her home environment, her husband assisted her during walking and performed tasks such as meal preparation, and her daughter, who lived nearby, visited once a week to help with housework. The primary objective was to enhance her indoor mobility and physical function, with the aim of reducing her husband's caregiving responsibilities. Despite a thorough investigation into the potential causes of her elevated inflammatory and oxidative stress markers, including her medical history of hypertension, heart failure, and lifestyle habits, no definitive explanation was found. Consequently, hydrogen inhalation therapy was introduced as an additional treatment modality along with the existing program. It is noteworthy that this decision was not made based on definitive evidence that hydrogen inhalation therapy was the optimal treatment; rather, it was based on the possibility that it might help reduce inflammation and oxidative stress. Moreover, due to her low albumin level and BMI, a dietitian was consulted to implement nutritional counseling and recommend a high-protein diet to both the patient and her family.

Walking was performed under close supervision, with her gait characterized by low foot clearance and a shuffling pattern. Additionally, marked trunk sway was observed in the frontal plane, and the TUG result indicated a potentially high fall risk [25-28]. Given the patient's history of heart failure and significant fluctuations in blood pressure before and after six-minute walks, it was presumed that her exercise tolerance would be low. Consequently, she participated primarily in group exercise sessions and individualized low-intensity whole-body exercise programs. Specifically, she used a recumbent bike (Pre Line; Sakai Medical Co., Ōnojō, Kyushu, Japan) and performed machine training (Compass Z series; Sakai Medical Co.) on leg extension, leg press, and chest press. Given her ability to report subjective symptoms of fatigue, exercise intensity was monitored such that the Borg scale would not exceed 11 ("light") and her heart rate, calculated by the Karvonen formula, would remain at or below 60% of maximum.

The hydrogen inhalation therapy involved the use of a hydrogen generator (Lita Air; WCJ Co., Ltd, Osaka, Japan) to produce hydrogen gas at a concentration of 3-4%, a level that has been confirmed to be safe in clinical trials and to influence hemodynamic parameters [29]. The patient inhaled hydrogen gas for approximately 60 minutes before beginning her individualized exercise program. Due to the absence of a control group, it is not possible to draw definitive conclusions regarding the effects of the intervention.

The intervention program was administered thrice weekly, aligning with the patient's scheduled daycare rehabilitation sessions, for a duration of three months. Each session encompassed approximately two hours of exercise, comprising group exercises and individualized programs. Of this time, approximately 30 minutes was designated for one-on-one interventions with a therapist. Group exercises were implemented to cultivate social connections and enhance motivation, while seated exercises utilizing TheraBands (Theraband, Akron, Ohio, United States) lasted for 30 minutes. 

The intensity of the individualized program was adjusted based on periodic assessments of vital signs and perceived fatigue. Initially, the patient frequently complained of fatigue and dyspnea, prompting incremental increases in the duration of group exercise and recumbent bike use. Exercise intensity was set around Borg scale 11 ("light") and approximately 40% of maximum heart rate by the Karvonen formula, with adequate rest intervals. Approximately 30 days after the intervention's commencement, the patient demonstrated the capacity to cycle for over 10 minutes on the recumbent bike. Consequently, the workload was methodically augmented, with the objective of attaining Borg scale 11 and 60% of maximum heart rate. By the 48th day, the patient had attained the ability to sustain over 15 minutes of exercise on the recumbent bike, thus facilitating the incorporation of machine training (leg extension, leg press, and chest press) into the regimen. No specific load was initially set; intensity was adjusted by extending exercise duration. Concurrently, she began arriving at the facility without using a T-cane, and based on the therapist's evaluation, she was deemed able to ambulate independently within the facility. Parallel bar exercises such as tandem walking and step exercises were introduced to further improve balance. Hand support was gradually reduced to increase balance demands. 

Patient education was initiated at the commencement of the intervention and encompassed the patient, her husband (the primary caregiver), and her daughter. The fundamental components of the patient education program comprised the following: (i) Nutritional counseling by a dietitian, (ii) Encouragement of as much daily exercise as possible at home (e.g., home exercise protocols), and (iii) Refraining from overexertion when feeling unwell. The nutritional guidance focused on explaining the need for weight gain, providing information on high-protein meals, and suggesting supplemental nutrition products. 

**Figure 1:**
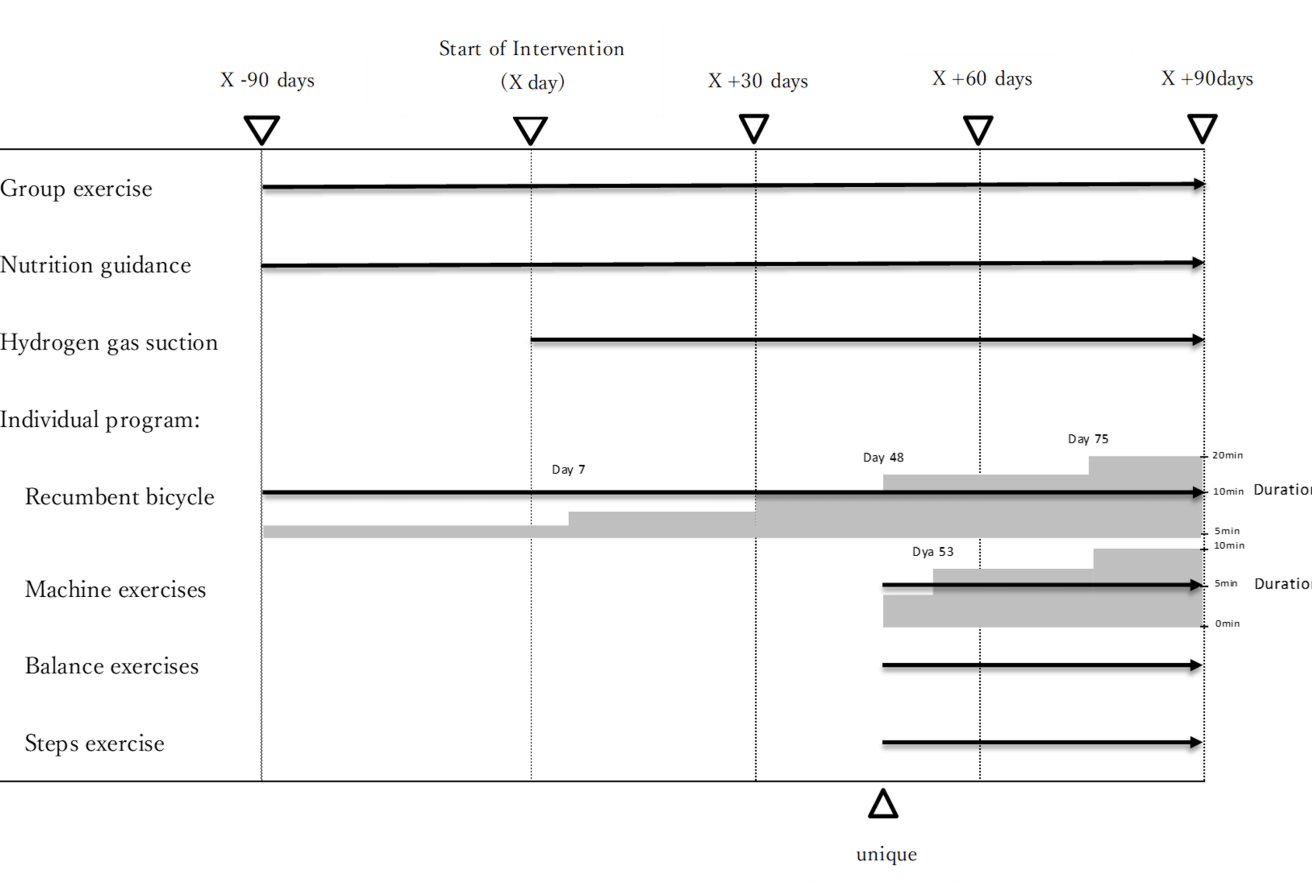
Progress of Intervention

Figure 1 illustrates the schematic overview of the multifaceted intervention protocol used in this collaborative study.

Post-intervention results

After three months of intervention, the patient showed significant improvement. Indoors, the patient was able to walk independently without aids. For outdoor walking on uneven terrain, the patient continued to use a T-cane but only required supervision. Family Caregiver Burden Scale (FCS) scores improved from 31/40 to 17/40, which is in the normal range. Improvements were in the areas of caregiver emotional burden, percentage of time devoted to caregiving, caregiver health, and anxiety about the future.

Physical Findings

Height remained at 152.4 cm, weight increased to 42.8 kg, BMI increased to 18.4 kg/m² and showed a slight improvement in body composition; on the HDS-R, the score was 26/30, with orientation, backward-pointing, and object recall subject to point reduction. Resting blood pressure was 114/72 mmHg and resting heart rate 68 beats/minute; after the six-minute walk test, blood pressure was 120/74 mmHg and heart rate 72 beats/minute with no complaints of breathlessness or fatigue.

Laboratory Findings

Post-intervention laboratory results showed CRP at 0.6 mg/dL, BNP at 40.7 pg/dL, IL-6 at 6.2 pg/mL, albumin at 5.3 g/dL, and 8-OHdG at 0.38 ng/mL.

Physiotherapy Assessment

Leg circumference increased to 32.6 cm. Knee extensor strength was grade 4 on manual muscle testing (MMT), grip strength was 18.5 kg left hand and 20.0 kg right hand, walking speed was 1.2 m/s for six minutes without T-cane, sit-to-stand test was completed in 13.6 seconds, which was unmeasurable at baseline. Skeletal muscle index was 5.8 kg/m². According to AWGS2019 criteria, the patient exceeded cut-off values for all parameters except the sit-to-stand test. The TUG test, performed at a comfortable pace, lasted 14.6 seconds without the T-cane (Table 1).

**Table 1:** Results of longitudinal evaluation “(+)” indicates the presence of symptoms (e.g., shortness of breath and fatigue); “(−)” indicates absence *Reference ranges may vary by laboratory. The listed values are commonly used benchmarks. HDS-R: Hasegawa Dementia Scale-Revised; X: start of intervention

Parameters	Reference Range*	X - 90 days	Pre-intervention Results (X day)	Post-intervention Results (X+ 90 days)
Physical Findings				
Stature		152.3 cm	152.4 cm	152.4 cm
Body Weight		40.4 kg	41.1 kg	42.8 kg
BMI		17.4 kg/m²	17.7 kg/m²	18.4 kg/m²
HDS-R	Typically (＞20)	23 / 30 score	25 / 30 score	26 / 30 score
Vital Signs				
Resting Blood Pressure	90 - 130 / 60 - 80 mmHg (typical)	110 / 70 mmHg	104 / 74 mmHg	114 / 72 mmHg
Resting Pulse Rate	60 - 100 /min (typical)	60 /min	62 /min	68 /min
Blood Pressure After 6-minute Walk Speed Test	90 - 130 / 60 - 80 mmHg (typical)	130 / 82 mmHg	128 / 82 mmHg	120 / 74 mmHg
Pulse Rate After 6-mute Walk Speed Test	60 - 100 /min (typical)	88 /min	84 /min	72 /min
Shortness of Breath and Fatigue	Typically ( - )	( + )	( + )	( - )
Laboratory Findings				
C-reactive Protein	＜0.3 mg/dL	1.4 mg/dL	1.2 mg/dL	0.6 mg/dL
Brain Natriuretic Peptide	＜100 pg/dL	43.0 pg/dL	43.2 pg/dL	40.7 pg/dL
Interleukin-6	＜4.0 pg/mL (approximate)	8.8 pg/mL	8.4 pg/mL	6.2 pg/mL
Albumin	3.8 - 5.2 g/dL	3.5 g/dL	3.6 g/dL	5.3 g/dL
8-Hydroxy-2′-deoxyguanosine (8-OHdG)	＜0.40 ng/mL (approximate)	0.44 ng/mL	0.46 ng/mL	0.38 ng/mL
Physical Therapy Assessment				
Lower Leg Circumference	Typically（＞33cm; female）	31.0cm	31.2cm	32.6cm
Knee Extension (MMT)		3 level	4 level	4 level
Grip Strength Left/Right	Typically（＞18 kg; female）	16.0 kg / 16.2 kg	16.5 kg / 17.2 kg	18.5 kg / 20.0 kg
6-minute Walk Speed Test (comfortable)	Typically（＞1 m/sec）	0.6 m/sec（T-cane）	0.6 m/sec（T-cane）	1.2 m/sec（unique）
Sit to Stand-5 (SS-5)	Typically（＜12 sec ）	-	-	13.6 sec
Skeletal Muscle Index	Typically（＜5.7 kg/m²; female）	5.4 kg/m²	5.5 kg/m²	5.8 kg/m²
Timed Up-and-Go test (comfortable)	＜7.91sec(Normal)	18.8 sec（T-cane）	17.4 sec（T-cane）	14.6 sec（unique）
Family Caregiver Burden Scale		35/40（heavy group）	31/40（heavy group）	17/40点（normal group)

## Discussion

This report described the case of a female patient in her early 80s who began day-care rehabilitation following a fall at home. Initial evaluation indicated suspected sarcopenia, elevated inflammatory and oxidative stress markers, and poor nutritional status. Her husband, the patient's primary caregiver, reported a high caregiving burden, necessitating interventions to alleviate physical and psychological strain. The subsequent sections will address the effectiveness and mechanisms of the multifaceted interventions for this patient.

Significance of suspected sarcopenia and its diagnosis

Sarcopenia, characterized by reduced skeletal muscle mass and strength associated with aging, is closely linked to declines in physical function, increased fall risk, and reduced quality of life [30]. According to the AWGS2019 criteria for Asian populations, diagnosis involves evaluating muscle mass, muscle strength, and physical performance. The patient exhibited low grip strength, low SMI, and reduced walking speed, findings consistent with suspected sarcopenia. Sarcopenia has significant implications for independence and activities of daily living (ADL) in older adults. Muscle weakness increases fall risk and can lead to fractures or extended bed rest [31]. Additionally, skeletal muscle contributes to whole-body metabolism and immune function, and sarcopenia is associated with poorer outcomes in chronic diseases and an increased risk of hospitalization [32]. Initially, inpatient treatment was considered for this patient; however, she and her family elected to receive care through day-care rehabilitation services. Consequently, a multifaceted approach involving multiple professional disciplines and family members was implemented. After a three-month intervention, improvements in physical function, inflammatory markers, and nutritional status were observed.

Relationship between inflammation, oxidative stress, and sarcopenia

This patient presented with elevated CRP and IL-6 levels. IL-6 is a specific type of cytokine, which is a protein that plays a crucial role in the body's immune system. Elevated levels of IL-6 have been associated with sarcopenia [33]. This condition is believed to be a result of an inflammatory response in the body. Increased levels of inflammatory cytokines, such as IL-6, have been shown to accelerate the breakdown of muscle protein and suppress muscle protein synthesis, leading to muscle weakness [34]. 8-OHdG, on the other hand, is a biomarker that indicates oxidative damage to DNA and is a sign of oxidative stress [35]. Oxidative stress can compromise the function of mitochondria, which are essential for energy production in muscle cells, leading to muscle atrophy. These findings underscore the importance of early intervention in this patient's case.

Resistance exercises stimulate muscle protein synthesis and are recognized as effective in increasing muscle mass and strength [36]. The gradual introduction of machine-based exercises (leg extension, leg press, chest press) with incremental increases in resistance appears to have been appropriate for this patient.

The incorporation of a recumbent bike as an aerobic exercise modality has been shown to enhance cardiorespiratory function and exercise tolerance [37]. Moreover, the implementation of aerobic exercise at suitable intensity levels is a safe practice even for older adults with a history of heart failure, with the potential to improve cardiac function over time [38]. In the case of the patient in question, who had a medical history of heart failure and exhibited significant fatigability, this approach was deemed suitable.

Balance training, encompassing tandem walking and step exercises within parallel bars, has been shown to enhance dynamic balance and mitigate the risk of falls [39]. This is likely to have contributed to the observed improvements in gait parameters and reduced TUG times. However, the patient continued to demonstrate occasional toe catches on uneven surfaces, suggesting the need for additional gait training in outdoor settings and more advanced performance-oriented training in the future. 

Effects of hydrogen inhalation therapy 

Hydrogen gas has been shown to selectively scavenge reactive oxygen species, including hydroxyl radicals and peroxynitrite [40], thereby mitigating oxidative stress and suppressing inflammation. Although the patient exhibited a decrease in CRP, IL-6, and 8-OHdG levels following the intervention, it is challenging to ascertain the specific contribution of hydrogen inhalation therapy, given that the patient also received exercise therapy and nutritional interventions. Future research employing a control group or alternative study designs (e.g., applied behavior analysis (ABA) design) is necessary to more definitively ascertain the therapy's standalone effects although no physical effects have been reported. Molecular hydrogen is very fine and diffuses easily. Therefore, it is important to note that the hydrogen gas must be measured and controlled by a technician.

Effects of nutritional intervention 

Older adults often exhibit decreased sensitivity to anabolic stimuli, necessitating adequate protein intake to maintain or increase muscle mass [41]. This patient received dietary guidance aimed at a high-protein diet, which may have contributed to improvements in albumin, body weight, and BMI. In addition, the patient and her family were informed of the importance of micronutrients such as vitamin D, which has been associated with a reduced fall risk [42], and essential amino acids like leucine, which has been shown to stimulate muscle protein synthesis [43]. This information, in conjunction with the patient's home exercise program, is likely to promote further nutritional improvements.

Patient education and family involvement 

Home exercise programs have been reported to maintain rehabilitation gains and increase daily activity levels [44]. The patient was instructed to perform bodyweight exercises at home and used a calendar to record her adherence to the exercise program. She reported performing the exercises almost daily between daycare sessions, which likely aided in her functional improvements. Caregiver education and support systems are also crucial for reducing caregiver burden [45]. The patient's husband and daughter both received instruction regarding nutrition and exercise, fostering greater understanding and cooperation in caregiving. Following the intervention, the patient demonstrated significant improvements in mobility, which enabled her to walk independently indoors. Consequently, her husband's FCS score decreased from 31 to 17, indicating a shift into the normal range [46]. It is noteworthy that enhanced physical function has been associated with the alleviation of psychological stress experienced by caregivers [47]. Furthermore, the reduction in inflammation and oxidative stress may have contributed to muscle maintenance and strength gains by decreasing protein breakdown [48].

Observations and future directions

In summary, the present study involved the concurrent administration of multiple interventions (exercise therapy, nutritional therapy, and hydrogen inhalation therapy). The observed improvements can be attributed to the synergistic effects of comprehensive, interdisciplinary interventions, an approach deemed essential for geriatric care [49]. Ongoing multidisciplinary collaboration will be essential to further enhance physical function and reduce the burden of caregiving. In terms of future directions, the clinical application of hydrogen inhalation therapy represents a relatively novel field, necessitating further research to ascertain its efficacy and safety. While the present study yielded encouraging results, the need for large-scale randomized controlled trials and studies that directly compare combined interventions with single interventions is paramount to further strengthen the evidence base. Additionally, while the intervention duration in this study spanned three months, a sustained effort will be required to maintain and further enhance outcomes related to sarcopenia [50]. Longitudinal follow-up may reveal strategies to maintain treatment efficacy and prevent recurrence. It is also important to note that this study was only descriptive in its representation and did not assess other variables that promote rehabilitation effectiveness, such as caregivers and psychological aspects of the cases. Therefore, a detailed study in this regard is warranted in the future.

## Conclusions

Following a three-month multimodal intervention involving exercise therapy, nutritional guidance, and hydrogen gas inhalation, the patient demonstrated notable enhancements in grip strength, walking speed, and inflammation and oxidative stress markers (CRP, IL-6, 8-OHdG). Concurrently, caregiver burden, as gauged by the FCS, diminished from "severe" to the "normal" range. The findings of this case study suggest preliminary evidence that could be further explored in future studies, potentially serving as a preliminary report for larger-scale investigations and causal inferences.
